# Satisfaction and associated factors among psychiatry service users at Amanuel mental specialized hospital. Addis Ababa, Ethiopia

**DOI:** 10.3389/fpsyt.2022.952094

**Published:** 2022-09-15

**Authors:** Esayas Kibrom, Zebiba Naser, Merga Seyoum, Atakilit Mengesha, Kemeria Adem, Deribe Bekele Dechasa, Henock Asfaw

**Affiliations:** ^1^Research and Training Department, Amanuel Mental Specialized Hospital, Addis Ababa, Ethiopia; ^2^School of Nursing and Midwifery, College of Health and Medical Science, Haramaya University, Harar, Ethiopia

**Keywords:** satisfaction, psychiatric service, psychiatric patient, Ethiopia, mental health

## Abstract

**Background:**

Patient service satisfaction is the central point for the health system. Worldwide, around 450 million people suffer from mental and behavioral disorders. Clients who are dissatisfied with the service will miss appointments, abandon the treatment plan, and eventually relapse from the illness. Despite improved access to health services, the satisfaction from health institution services was decreasing from time to time and there is little information on health service consumer satisfaction.

**Objective:**

To assess the satisfaction and associated factors among psychiatric service consumers at Amanuel Mental Specialized Hospital, Addis Ababa, Ethiopia, 2021/2022.

**Methods and materials:**

An institutional-based cross-sectional study was conducted among 420 psychiatric service users from December 15 to January 16, 2021/2022. Systematic random sampling was used. A face-face interview technique and chart review were used to collect the data and a standard and validated tool called the mental health service satisfaction scale (MHSSS) was used to measure satisfaction. The collected data was entered into EPI info version 7 and exported to SPSS version 22 for analysis. The binary logistic regression model was used to analyze the data and bivariable and multivariable logistic regression analyses were conducted to identify associated factors with satisfaction. The level of significance was reported at *P* < 0.05.

**Results:**

The study showed that the magnitude of patient satisfaction was 63.3% [95% CI 58.3–67.9%). Sex (AOR = 1.7, 95% CI (1.072–2.88)], educational status (AOR 4.2, 95% CI 1.64–1.8), residency [AOR = 1.8, 95% CI (1.098–3.19)], distance from the hospital [AOR 0.56, (0.34–0.93)] were significantly associated with patient satisfaction at *p* < 0.05.

**Conclusion:**

The magnitude of patient satisfaction was high. The study showed that Sex, educational status, residence, and distance from the hospital were significantly associated with satisfaction. Prioritizing care for female patients and those coming from a distance is necessary. More ever, it is preferable to routinely and continually monitor healthcare facilities so that timely feedback can be given and problems that affect patient satisfaction can be resolved.

## Introduction

Patient satisfaction is the central point of measuring the health system (1, 2). It can be defined as the client's level gratification to a different psychiatric service offered by the health care system during health facility visits. Psychiatry patients evaluate the service providers with their subjective views on finding solutions to the problems they encounter. Service users compare the service with their experience and presumed expectations and consumer satisfaction is playing huge and important role in determining service quality reforms. However, finding an accepted meaning for quality of service is difficult to find (3). One of the desired results for mental health providers is patient satisfaction. It has been a major contributor to results in the measurement and enhancement of the quality of care for the mental health system (4).

According to WHO reports, the burden of mental disorders has risen significantly over the past decade and regular assessment of service Satisfaction is a key element in detecting problems that arise in day-to-day work that helps the health care facility find a system that can provide quality of service for the clients, and it is the cornerstone of the health care system. More than 450 million people suffer from behavioral and mental disorders worldwide annually, but there is not enough attention given in developing countries (5). Giving attention to mental illness and providing quality service is very necessary to address the population's problems (6).

Those who are dissatisfied with the service become noncompliant with the treatment, miss the appointment days, leave the treatment against medical advice, and it increases the relapse rate of the illness, so those individuals who face these problems are going to be unhealthy, unproductive, and could be a burden to the family, to the community, and the country (7).

Utilizing available resources effectively for service satisfaction can be a bridge to close the gap since the mental health sector lacks sufficient fundraising to maintain the system (8). The psychiatric patients' perception of the quality of service has an important role in providing quality care to the clients. Health facilities can retain their clients to recover from illness and improve the wellbeing of clients in the existing health care system by providing quality service (9).

Providing quality service should be a health facility goal that can be exercised and that can be achieved to the result of satisfying clients' needs, maintaining healthy individuals' health, and providing the country with active and productive people.

In recent decades, addressing patient satisfaction with the service has gained more concern and acceptance. Studies have also been carried out, and the required accurate and validated tools have been developed. The quality of service is influenced by different factors related to the clinical service's patient satisfaction, such as drug availability, service cost, neatness of the compound, quality of service, and service opening hours. However, quality of service can be provided by good communication skills with the clients that enable effective management as well as address clients' problems in getting the service to enable the service provider to give a convenient solution for patient satisfaction (10).

Additionally, because patient demands and attitudes toward services might fluctuate, it will be crucial for researchers to use this study's findings as a baseline for future assessments of this study area and other patient satisfaction-related study areas.

## Methods and materials

### Study area and period

This study was conducted at Amanuel Mental Specialized Hospital in Addis Ababa. It is one of the oldest hospitals, established in 1930 E.C during the Ethio-Italian war, and it is the only mental hospital in Ethiopia. It is located in the western part of Addis Ababa in Addis Ketema Sub-city, kebele 08. The hospital is working on increasing the efficiency and effectiveness of the services to make itself the center of mental health care excellence by providing core mental health clinical services, conducting research and training, and other administrative services. An average of 9,662 people visited outpatient departments each month, and 370 inpatients were available. The hospital has 300 beds that serve all types of mental disorders. The hospital has 17 OPDs. The study was conducted from December 15 to January 16, 2021/2022.

### Study design and population

An institutional-based quantitative cross-sectional study design was employed. All patients who were getting treatment and who were in remission at Amanuel Mental specialized hospital and who were available at the time of data collection and whose age was 18 years or older were included. Those who were severely ill and unable to communicate were excluded from the study.

### Sample size determination and sampling procedure

The optimum number of samples required for the study was estimated using a single population proportion formula considering the following assumptions: At a margin of error of 5%, at 95% CI, the magnitude of satisfaction was taken from a study done in St Paul's Hospital Millennium Medical College in Ethiopia, which was 50.3% (11), and the non-response rate was 10%. The final calculated sample size was 422. The sample for the second specific objective is determined by using the Epi Info version 7 by considering the factors that were significantly associated with satisfaction at (*p* = 0.05), a two-sided confidence level of 95% and margin of error of 5%, and power=80 percent, and a ratio of exposed to unexposed of 1:1. To get enough sample size we used the factor that gave us a large sample size and accordingly sample size was 330 and 10% non-response rate was added. By adding 10% to 330 it gave us 363 which is <422. Since 422 is larger than 363, thus the final sample size was 422.

A stratified sampling technique was used to select samples from inpatient and outpatient departments. Then systematic random sampling was used to select sampling units from each ward and outpatient department. The selection interval was that participants selected every 23rd interval. The first individual was selected by the lottery method.

### Data collection tools and procedures

Data on psychiatric patient satisfaction was collected through interviews using a standardized client satisfaction questionnaire (CSQ-8), developed for use in mental health programs, where the tools have eight individual items and the rank order is the same for all questions, with four options ranging from 1 = “Poor, 2 = Fair, 3 = Good and 4 = Excellent.” In our study, poor and fair are regarded as dissatisfied, and good and excellent are regarded as satisfied. Information on the clinical factors was collected by the reviewing patient's chart. Sociodemographic and other variables were assessed using structured questionnaires developed from different studies in the literature (12). Social support was also measured by the Oslo Social Support Scale (OSSS)—it is a three-item scale with a Cronbach's alpha of 0.75 and has a range value of 3–14, further categorized as follows; poor support (3–8), moderate support (9–11) and strong support (12–14) (13).

### Data quality control

To assure the data quality, high emphasis was given to the data collection instrument. The training was given to data collectors and supervisors for 2 days. Before starting the actual survey, the questionnaire was pretested on 21 individuals. The findings of the pretest help to revise and adapt the questionnaire and estimate the time required for the interview. The data collectors were supervised daily, and the filled questionnaires were checked properly by the supervisor and principal investigator. If a problem arose, a solution was provided by timely discussions with the supervisor and data collectors.

### Operational definitions

The client satisfaction questionnaire (CSQ-8), is eight individual items tool used to assess psychiatry patient satisfaction it has four response options ranging from 1 = “Poor, 2 fair, 3= Good, and 4 = Excellent.” Poor and fair are regarded as dissatisfied and good and excellent are regarded as satisfied. The score ranges from 8 to 32 a score <16 was regarded as dissatisfied and 16 and above was regarded as satisfied (14).

Social support was also measured by the OSSS and it is a three-item scale and has a range value of 3–14, further categorized as follows; poor support (3–8), moderate support (9–11), and strong support (12–14) (13).

### Data processing and analysis

The data was entered by Epi-data version 3.1 to minimize data entry errors and then exported to SPSS version 22.00 for analysis. Descriptive statistics such as text, percentages, graphs, and tables for categorical data and calculated mean and standard deviation for continuous variables were done. All variables that had a *P*-Value of < 0.2 in bivariate analysis were entered into multivariate analysis. The strength of the association was determined by using an odds ratio with 95% CI and a *P*-value <0.05 in multivariate analysis was considered a statistically significant association. The logistic regression model's fitness was tested using Hosmer and Lemeshow, and the result was a fit of 0.75. The multicollinearity was checked by the variance inflation factor and the result was 1.2.

### Ethical consideration

The Ethical Review Committee (ERC) of Saint Amanuel Mental Specialized Hospital gave their approval. A formal letter of permission from Saint Amanuel mental specialized hospital was received and submitted to the medical directorate of Saint Amanuel mental specialized hospital. Participants in the study provided written informed consent. By eliminating personal identifiers, confidentiality was maintained.

## Results

### Socio-demographic characteristics of patients

Of a total of 422 patients who were asked to participate in the study, 420 (99.5%) were fully interviewed. The mean age of the participants was 38.8% (±12.8), with ages ranging from 18 to 80 years. Among the respondents, nearly one-third 161 (38.3%) of the respondents were within the age range of 31–40 years. About 247 (58.8%) were female, more than half, 287 (68.3%) were Christians, and 128 (30.5%) were married. The educational status of participants indicated that about 111 (26.4%) of them attended high school. Regarding occupation, about 122 (29.0%) of participants were jobless. More than half of the participants (69.0%) came from rural areas. More than half, 290 (69.0%), got free service. The living status of most of the patients who live with their families is 170 (40.5%). More than half of the participants come from a distance of 600 km up to 1,000 km (290) (69.0%) (Table 1).

**Table 1:** Distribution of participants by socio-demographic factors visiting out-patient and in-patient service at Amanuel Mental Specialized Hospital Addis Ababa, Ethiopia, 2021 (*n* = 420).

**Variable**	**Category**	**Frequency (*N* = 420)**	**Percent (%)**
Age	18–30	139	33.1
	31–40	161	38.3
	41–50	72	17.1
	>51	48	11.4
Sex	Male	247	58.8
	Female	173	41.2
Religion	Orthodox	139	33.1
	Muslim	133	31.7
	Protestant	89	21.2
	Catholic	59	14.0
Marital status	Single	128	30.5
	Married	116	27.6
	Divorced	55	13.1
	Separated	66	15.7
	Widowed/widower	55	13.1
Education status	Illiterate	123	29.3
	Primary	108	25.7
	High school	111	26.4
	Diploma	48	11.4
	Degree and above	30	7.1
Occupational status	Employed	42	10.0
	Private Business	50	11.9
	Daily labor	45	10.7
	Farmer	80	19.0
	Jobless	122	29.0
	Student	50	11.9
	Pensioned	31	7.4
Residency	Urban	130	31.0
	Rural	290	69.0
Service getting	Free	290	69.0
	With charge	130	31.0
Living status	Alone	79	18.8
	With parent	111	26.4
	With family	170	40.5
	With other	60	14.3
Distance from the Hospital	<600 km	179	42.6
	600–1,000 km	241	57.4

### Clinical related characteristics

Regarding the clinical characteristics of the respondents, 111 (26.4%) were diagnosed with schizophrenia and 89 (21.2%) were bipolar patients. Among the participants, 147 (35.0%) had more than 5 years of follow-up. Participants reporting little availability of medication were 163 (38.8%). Half of the participants reported that the waiting area condition was fair (Table 2).

**Table 2:** Description of clinical factors among service consumers in out-patient and in-patient service at Amanuel Mental Specialized Hospital Addis Ababa, Ethiopia, 2021 (*n* = 420).

**Variable**	**Category**	**Frequency (*N* = 420)**	**Percent (%)**
Types of mental illness	Schizophrenia	111	26.4
	Bipolar	89	21.2
	Depression	79	18.8
	Anxiety	45	10.7
	Epilepsy	50	11.9
	Others	46	11.0
Duration of follow up	1 month up to 6 months	77	18.3
	7 months−1 year	84	20.0
	1 year−5 years	112	26.7
	>5 years	147	35.0
Availability of medication	No	73	17.4
	Little	163	38.8
	Available	136	32.4
	Very available	48	11.4
Waiting area condition	Poor	40	9.5
	Fair	210	50.0
	Comfortable	145	34.5
	Very comfortable	25	6.0

### Psycho-social related characteristics

Regarding social support, 197 (46.9. %) had moderate social support, 124 (29.5%) had strong social support and 99 (23.6%) had poor social support.

### The magnitude of patient satisfaction

**Figure 1:**
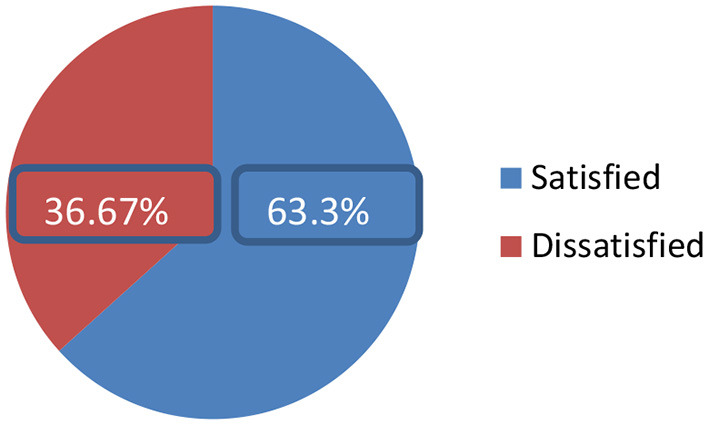
Magnitude of satisfaction among service consumers in out-patient and in-patient at Amanuel Mental Specialized Hospital Addis Ababa, Ethiopia 2021 (*N* = 420).

Concerning the magnitude of patient satisfaction, 261 (63.3%) (95% CI 58.3% to 67.9%) were satisfied and 151 (36.67%) (95% CI 32.1 to 41.7%) were dissatisfied (Figure 1).

### Factors associated with patient satisfaction

In bivariable binary logistic analysis, variables including sex, educational status, occupation, residence, living status, duration of the illness, and distance from the hospital were found to have *P* < 0.2. These variables fulfilled the minimum requirements for further multivariable binary logistic regression.

From multivariable binary logistic regression only; male sex, rural residence, and having no formal education were significantly associated with patient satisfaction at a *P* < 0.05.

The odds of being satisfied with psychiatry service among male patients were 1.76 times the odds of female patients (AOR = 1.76; 95% CI = 1.07–2.88). The odds of being satisfied with psychiatry service among rural resident patients were 1.88 times the odds of urban resident patients (AOR = 1.88; 95% CI = 1.09–3.19). The odds of being satisfied with psychiatry service among illiterate patients were 2.21 times the odds of patients who had a degree and above (AOR = 2.21; 95% CI = 1.64–4.81). Patients who came from a distance of 600–1,000 km were 44% less likely to be satisfied with psychiatry service compared to patients who came from a distance of <600 km (AOR = 0.56; 95% CI = 0.34–0.93) (Table 3).

**Table 3:** Bivariable and multivariable logistic regression analysis showing the association between factors and satisfaction among service consumers in out-patient and in-patient at Amanuel Mental Specialized Hospital Addis Ababa, Ethiopia 2021 (*n* = 420).

**Explanatory variables**	**Category**	**Satisfaction**		**COR, (95%CI)**	**AOR, (95%CI)**	***P*-value**
		**Satisfied**	**Dissatisfied**			
Sex	Male	170	77	1.77 (1.18–2.65)	1.76 (1.07–2.88)	0.025^*^
	Female	96	77	1	1	
Educational status	Illiterate	101	22	3.52 (1.49–8.27)	2.21 (1.64–4.81)	0.003*
	Primary school	59	49	0.92 (0.41–2.08)	1.16 (0.48–2.79)	0.37
	High school	61	50	0.93 (0.42–2.10)	1.18 (0.49–2.85)	0.36
	Diploma	28	20	1.07 (0.43–2.69)	1.13 (0.42–3.03)	0.47
	Degree and above	17	13	1	1	
Occupation	Employed	23	19	1	1	
	Private business	33	17	1.60 (0.69–3.72)	1.13 (0.44–2.89)	0.35
	Daily labor	27	18	1.23 (0.53–2.90)	1.09 (0.41–2.90)	0.27
	Farmer	52	28	1.534 (0.72–3.28)	1.07 (0.44–2.63)	0.31
	Student	75	47	1.32 (0.65–2.67)	1.51 (0.66–3.42)	0.43
	Jobless	37	13	2.35 (0.98–5.65)	1.86 (0.69-4.98)	0.51
	Pensioned	19	12	1.31 (0.51–3.36)	0.95 (0.32–2.86)	0.28
Residence	Urban	70	60	1	1	
	Rural	196	94	1.78 (1.17–2.73)	1.88 (1.10–3.19)	0.021*
Living status	Alone	71	29	2.08 (1.11–3.90)	1.91 (0.94–3.85)	0.28
	With partner	57	34	1.42 (0.76–2.66)	1.59 (0.78–3.22)	0.27
	With family	98	57	1.46 (0.83–2.56)	1.54 (0.83–2.83)	0.18
	With other	40	34	1		
Distance of hospital	<600 km	123	56	1		
	600–1,000 km	143	98	0.66 (0.44–0.99)	0.56 (0.34–0.93)	0.01*
Duration of follow up	1 up to 6 months	42	36	1		
	7 months up to 1 year	38	23	1.41 (0.72–2.80)	1.35 (0.63–2.93)	0.13
	1 up to 5 year	66	37	1.52 (.83–2.78)	1.35 (0.69–2.64)	0.11
	>5 year	120	58	1.77 (1.03–3.06)	1.82 (0.97–3.40)	0.26

* Significant at P < 0.05, COR, Crude Odd Ratio; AOR, Adjusted Odd Ratio; CI, Confidence Interval, 1, Reference.

## Discussion

In this study, patient satisfaction in inpatient and outpatient psychiatric settings was assessed. Of the study participants, 63.3% were satisfied, while 36.67% were dissatisfied which is consistent with the study done at Dessie Referral Hospital which was 61.3% (7). However, the proportion of patients who were satisfied with the general mental healthcare service was lower than in other studies, such as 91% in the USA (15), 81% in Germany (16), Romania (78%) (17), and China (77.1%) (18). The disparity could be attributed to the use of different measurement tools (while the list/white list tool used in Germany), different data collection methods (self-administered questionnaire used in Romania), differences in sociocultural factors, availability of the materials for the provision of qualified service and larger sample size in Romania. Conversely, the result was higher than the findings from Jimma 50.3% (19), Egypt (50%) (20), and 54.6% in Switzerland (21). The difference could be attributable to a difference in setting and study design in which the Egypt study was done at a national level and a prospective study design was done in Switzerland. The tool used to assess satisfaction in Jimma was 24 items questionnaires but in our study, we used the CSQ-8 questionnaire (19).

Regarding the factors associated with patient satisfaction, the odds of being satisfied with psychiatry service among male patients was 1.76 times the odds of female patients. The results are supported by studies done at saint Paul's Hospital (11), Dessie Referral Hospital (7), Jimma Hospital (19), Japan (22), Qatar (23), and Switzerland (21). This could be due to women in Ethiopian society being more conservative, and they receive less exposure to the general population. Less patient satisfaction among female patients could be attributed to this culture. Male are more involved in the decision-making process than females.

The odds of being satisfied with psychiatry service among illiterate patients were 2.21 times the odds of patients who had a degree and above. This finding coincides with studies done in Ethiopia (14), India (24), and Pakistan (19). This demonstrates that patients with comparatively lower levels of education have lower expectations and are less critical when evaluating the services received. These can leave individuals feeling more satisfied.

The odds of being satisfied with psychiatry service among rural resident patients were 1.88 times the odds of urban resident patients, and it is consistent with studies done at Dessie Referral Hospital (7). A possible explanation for this study seems to be that patients from rural residences come with fewer expectations from the service and less access to information about medical services and quality of care.

Patients who came from a distance of 600–1,000 km were 44% less likely to be satisfied with psychiatry service compared to patients who came from a distance of <600 km. This finding was supported by a study done in Ethiopia (19). The possible reason could be that coming from a long distance for psychiatric services led them to suffer, which created dissatisfaction because patients couldn't afford payment for transportation and hotel costs.

### The strength and limitations of the study

The response rate in this study was high, which helped to reduce the probability of non-response. To reduce bias, we employed a standardized and pre-tested questionnaire. Since this study used a cross-sectional design, it was challenging to demonstrate a temporal link between dependent and independent variables and respondents showing their experience this time and sometimes ago, this may introduce recall bias as well.

## Conclusion

In this study, the satisfaction level of patients receiving mental health services at Amanuel Mental Specialized Hospital was found to be high. Those who were male, illiterate, rural residents, and came from far away were found to have a statistically significant association with satisfaction.

### Recommendation

Prioritizing care for female patients and those coming from a distance is necessary. More ever, it is preferable to routinely and continually monitor healthcare facilities so that timely feedback can be given and problems that affect patient satisfaction can be resolved.

## Data availability statement

The original contributions presented in the study are included in the article/supplementary material, further inquiries can be directed to the corresponding author.

## Ethics statement

The studies involving human participants were reviewed and approved by the Ethical Review Committee (ERC) of Saint Amanuel Mental Specialized Hospital. The patients/participants provided their written informed consent to participate in this study.

## Author contributions

EK, ZN, MS, AM, and KA conceived the research, framed the methods, did the analysis, and wrote the final paper. HA and DBD participated in writing the manuscript, framing the method, and write-up. All the authors read and agreed on the final manuscript.

## Conflict of interest

The authors declare that the research was conducted in the absence of any commercial or financial relationships that could be construed as a potential conflict of interest.

## Publisher's note

All claims expressed in this article are solely those of the authors and do not necessarily represent those of their affiliated organizations, or those of the publisher, the editors and the reviewers. Any product that may be evaluated in this article, or claim that may be made by its manufacturer, is not guaranteed or endorsed by the publisher.

## Abbreviations

AOR: Adjusted Odds Ratio
CI: Confidence Interval
CSQ: Client Satisfaction Questionnaire
MHSSS: Mental Health Service Satisfaction Scale
OPD: Out Patient Department
OR: Odds Ratio
WHO: World Health Organization.
